# δ-tocotrienol suppresses the migration and angiogenesis of trophoblasts in preeclampsia and promotes their apoptosis via miR-429/ ZEB1 axis

**DOI:** 10.1080/21655979.2021.1923238

**Published:** 2021-05-18

**Authors:** Mei Shi, Xiuyun Chen, Hui Li, Lixia Zheng

**Affiliations:** a Department of Delivery Room, Jinan Second Maternal and Child Health Hospital, Jinan City, Shandong Province, China; b Department of ICU, Jinan Second Maternal and Child Health Hospital, Jinan City, Shandong Province, China; c Department of VIP Ward, Jinan Second Maternal and Child Health Hospital, Jinan City, Shandong Province, China

**Keywords:** δ- tocotrienol, miR-429, ZEB1, preeclampsia

## Abstract

Preeclampsia (PE) is a severe medical disorder during pregnancy and there has been controversy about the effects of vitamin E on PE. This research intended to explore if δ-tocotrienol (δ-TT), an isomer of vitamin E, could impact PE. Preeclamptic and normal placentas were obtained and total RNA was extracted. The expression of different genes was analyzed through quantitative real-time polymerase chain reaction (qRT-PCR) and Pearson correlation analysis was conducted. After that, HTR-8/SVneo cells (human trophoblasts) were chosen and they were subjected to δ-tocotrienol treatment and then Cell Counting Kit-8 was used to test cell viability. To assess the effects of δ-TT on trophoblasts, wound healing assay and Transwell invasion assay were performed. How miR-429 interacts with ZEB1 was examined via dual luciferase reporter assay. Also, protein expression was evaluated via Western blotting. Our results have shown that δ-TT can impair the viability of trophoblasts and induce their apoptosis. Additionally, it can repress the growth, migration, epithelial-mesenchymal transition (EMT), invasion and angiogenesis in trophoblasts. Mechanistically, δ-TT exerts these effects on trophoblasts via downregulating miR-429 and upregulating ZEB1. Furthermore, miR-429 can bind ZEB1 directly. Clinical sample analysis has revealed that miR-429 expression in preeclamptic placenta is higher than that in normal placenta, but ZEB1 expression in preeclamptic placenta is downregulated. Also, there is a negative association between miR-429 and ZEB1 expression in preeclamptic placentas. These discoveries imply that δ-TT may be hazardous to pregnancy and should not be used in preeclamptic patients. In addition, targeting miR-429 might treat PE.

## 1. Introduction

Preeclampsia (PE) is a medical disorder that occurs during pregnancy and the main characteristics of this disease include hypertension and proteinuria in excess of 300 mg/day [1]. This disease can affect 3–10% of pregnancies and the common risk factors of PE include obesity, chronic hypertension and pregestational diabetes [2]. It may deteriorate to a stage where maternal and fetal health can be put in danger [3]. The pathogenesis of this disease is quite complicated. In PE, trophoblasts have been found to become less invasive and migratory, and there is increased apoptosis of trophoblasts [4]. Many scientists believe that the malfunctioning of trophoblasts may contribute to PE, as they invade uterine wall during normal pregnancy and help to establish a low-resistance vascular network between mother and fetus [5,6]. Also, dysregulated trophoblasts can secrete the excessive amounts of soluble fms-like kinase 1 (sFlt-1), and this molecule can lead to endothelial dysfunction, thus increasing the risks of pulmonary edema and even stroke [7,8]. As a result, restoring the viability of trophoblasts and reducing their apoptosis are considered as the potential approach to treating PE.

Moreover, many studies have pointed out that in contrast to normal pregnancy, plasma vitamin E concentration during PE is significantly lower, implicating that vitamin E deficiency might contribute to PE [9,10]. Also, oxidative stress level during PE is much higher when compared to normal pregnancy [11], and vitamin E is regarded as a potent antioxidant chemical. Thus, many researchers propose that vitamin E supplementation during PE might alleviate or prevent this medical condition. Despite many studies, the role of vitamin E in PE and pregnancy remains inconclusive. Yong Guo et al. discovered that vitamin E supplementation could reduce the risk of the early onset of PE [12]. Nevertheless, the meta-analysis of randomized controlled trials revealed that the supplementation of vitamin E during PE did not show any preventative effects, but this would reduce the incidence of intrauterine growth restriction [13]. Another concern is that the intake of high-dose vitamin E is likely to increase the risk of low birth weight and neonatal jaundice, as vitamin E at high concentration can reduce the viability of trophoblasts [14,15].

It is worth mentioning that vitamin E has eight isomers and each isomer has different biological effects. For example, α-tocotrienol can protect neurons from stroke-induced damage [16]. Yet, β-tocotrienol has been found to exhibit proapoptotic effects on breast cancer cells [17]. Recently, δ-TT has attracted the attention from many scientists, as it has been shown to have anti-cancer effects. It was reported that δ-TT could sensitize the response of pancreatic cancer cells to gemcitabine by inhibiting NF-κB signaling [18]. Additionally, it could reduce the migration, invasion, EMT and angiogenesis of pancreatic ductal adenocarcinoma [19]. Although its anticancer effects have been well established, its influence on pregnancy and PE has not been explored.

Given the pro-apoptotic and anti-angiogenic effects of δ-TT on many cancers, we hypothesized that δ-TT might exert detrimental effects on trophoblasts. Thus, this research intended to study if δ-TT could affect the biological behaviors of trophoblasts and explore the underlying mechanism. The following is what we have discovered: (1) δ-TT induces the apoptosis of trophoblasts; (2) δ-TT impairs trophoblast’s migration, vasculogenic activity and epithelial-mesenchymal transition (EMT); (3) δ-TT exerts its proapoptotic, anti-migratory, anti-angiogenic and anti-EMT effects on trophoblasts through miR-429/ZEB1 axis; (4) miR-429 expression in preeclamptic placenta is higher than that in normal pregnant placenta. These results indicate δ-TT, an isomer of vitamin E, should be used with prudence in pregnancy and PE.

## 2. Materials and methods

### 2.1 Clinical specimens

This research was approved by the Ethical Committee of Jinan Second Maternal and Child Health Hospital. This study enrolled 46 women with normal pregnancy and 46 patients diagnosed with PE in our hospital from December 2017 to December 2019. They were Han Chinese people in origin and they did not receive any medical treatment before being admitted to our hospital. Apart from PE, they did not have any other diseases. Patients diagnosed with PE and those women with normal pregnancy enrolled in this study chose elective cesarean delivery without labor during their third trimester of gestation. In this research, PE was diagnosed when patient’s systolic blood pressure was more than 140 mmHg and/or diastolic blood pressure was more than 90 mmHg on 2 occasions at least 4 hours apart with their 24 h urine protein concentration ≥ 300 mg/day after the 20th week of gestation, but their proteinuria resolved before the 12th week postpartum. After we obtained their consent and they signed the informed written consent, blood samples and placentas were obtained from each participant. Blood samples were taken 1 day before elective cesarean section. Following elective cesarean section, placentas were instantly collected and washed by phosphate buffer saline. About 1 cm^3^ placenta fragments were dissected from each quadrant and the central part. Blood samples and tissue samples were stored at −80°C for further analysis. The clinical and pathological features of patients are shown in Table 1.

**Table 1.** Clinicopathological features of healthy pregnant women and women with PE

	Control (n = 46)	Preeclampsia (n = 46)	p Value
Maternal age (years)	29 ± 1.4	33.2 ± 6.4	0.21
Gestational age (days)	283.2 ± 4.6	236.4 ± 27.8	< 0.01
Birthweight (g)	3692.3 ± 584.3	2193.3 ± 882.3	< 0.01
BMI (kg/m^2^)	28.3 ± 2.5	31.5 ± 2.9	0.19
Proteinuria (g/24 h)	Not detected	2.9 ± 2.2	
SBP (mm/Hg)	120.3 ± 20.8	175.3 ± 18.7	< 0.01
DBP (mm/Hg)	88.2 ± 11.2	105.3 ± 11.4	< 0.01

### 2.2 Reagents

δ-TT was bought from MedChemExpress, USA (Cat. No: HY-122,778). It was dissolved in dimethyl sulfoxide (DMSO, Sigma-Aldrich, USA). The primary antibodies against cleaved caspase-3 (dilution: 1:1000, Abcam, UK; ab32042), cleaved caspase-9 (dilution: 1:1000, Abcam, UK; ab2324), cyclin D1 (dilution: 1:1000, Abcam, UK; ab16663), MMP-2 (dilution: 1:1000, Abcam, UK; ab92536), ZEB1 (dilution: 1:1000, Abcam, UK; ab203829), E-cadherin (dilution: 1:1000, Abcam, UK; ab40772), N-cadherin (dilution: 1:1000, Abcam, UK; 76,011), vimentin (dilution: 1:1000, Abcam, UK; 92,547) and GAPDH (dilution: 1:1000, Beyotechnology, China; AF1186) were used in this research.

### 2.2 Cells and cell culture

HTR-8/SVneo cells were bought from American Type Culture Collection (ATCC, USA). These cells were cultured in Dulbecco’s modified Eagle’s medium (DMEM, Sigma-Aldrich, USA) which contained 10% fetal bovine serum (Gibco, USA) and 1% penicillin/streptomycin (Beyotime, Shanghai, China). These cells were incubated at 37°C in a humid atmosphere containing 5% CO_2_.

### 2.3 Transfecting cells with plasmids and siRNAs

The following products were bought from Genomeditch (Shanghai, China): si-ZEB1, scramble siRNA for ZEB1 (si-NC), mimic negative control (NC), miR-429 mimic, inhibitor negative control (NC), and miR-429 inhibitor. Lipofectamine 3000 (Invitrogen, USA) was employed as the transfection reagent.

### 2.4 Quantitative real-time PCR (qRT-PCR)

We used Trizol reagent (Sigma-Aldrich, USA) to obtain total RNA, and miRNA was obtained by using Molpure Cell/Tissue miRNA Kit (Yeasen, Shanghai, China). In addition, mRNA was transcribed by using Hifair One Step RT-qPCR Probe Kit (Yeasen, Shanghai, China), yet we used TaqMan MicroRNA Reverse Transcription Kit (Invitrogen, USA) to transcribe miRNA. SYBR green was from Roche, Switzerland. U6 was the endogenous control for miR-429. The endogenous control for other primers was GAPDH. The primers in our experiments were listed in Table 2.

**Table 2.** Primer design

Gene	Primer 5’→3’
MiR429	F: ATGGGCGTCTTACCAGACAT
	R: GCGGATGGACGGTTTTACCA
ZEB1	F: GCTGTTTCAAGATGTTTCCTTCCA
	R: TTACACCCAGACTGCGTCAC
GAPDH	F: GGAGCGAGATCCCTCCAAAAT
	R: GGCTGTTGTCATACTTCTCATGG
U6	F: CTCGCTTCGGCAGCACA
	R: AACGCTTCACGAATTTGCGT

### 2.5 Cell viability and proliferation assay

To assess the viability and proliferation of HTR-8/SVneo trophoblast cells, Cell Counting Kit-8 (CCK-8) was purchased from Beyotime, Shanghai, China. HTR-8/SVneo cells (1ⅹ10^5^ cells/ml; 100 μL) were plated in 96-well plates and then they were incubated at 37°C. They were treated with δ-TT for 24 h to assess cell viability and for 72 h to evaluate cell proliferation. Following that, 10 μL of CCK-8 was added into each well at different time points. Next, these cells were cultured for 1 h. Finally, the absorbance value of each sample was examined at 450 nm.

### 2.6 Wound healing assay

After HTR-8/SVneo cells (3 ⅹ10^6^ cells/mL) were seeded onto 6-well plates and the monolayer of cells formed, the culture medium was removed and phosphate buffer saline (Sigma-Aldrich, USA) was used to wash the cells, after which a scratch was made on each well of plates with a 200 μL sterile pipette tip. Then, HTR-8/SVneo cells were treated with δ-TT for 24 h. Pictures were taken under a phase contrast microscope (Olympus, Tokyo, Japan) immediately and at 24 h. Scratch-covered and cell-covered area at 0 h and 24 h was calculated by Image-Pro Plus 7.0 (Media Cybernetics, USA), and then wound closure percentage was determined as previously described [20].

### 2.7 Dual luciferase reporter activity assay

Luciferase reporter vectors with wild-type (pGL3-ZEB1-WT) and mutant-type (pGL3-ZEB1-MUT) binding sites were purchased from Genomeditch, Shanghai, China. Initially, HTR-8/SVneo cells were plated onto 96-well plates at the density of 1 ⅹ10^5^ cells/mL. When their confluence grew to 60–70%, the luciferase reporter vectors and different miRNAs (miR-429 mimic, miR-429 mimic negative control, miR-429 inhibitor and inhibitor negative control) were co-transfected into HTR-8/SVneo cells respectively, according to manufacturer’s guideline. At 48 h after the transfection, the dual luciferase activity was examined by dual luciferase reporter assay system (Promega, USA), according to manufacturer’s instructions.

### 2.8 Western blotting

Proteins were isolated from HTR-8/SVneo cells by lysing them with radioimmunoprecipitation assay buffer on ice, and then their concentration was determined by Bicinchoninic Acid Kit (Beyotime, Shanghai, China). Next, proteins (30 μg per well) were loaded into sodium dodecyl sulfate polyacrylamide electrophoresis (SDS-PAGE) gel for electrophoresis. Then, proteins in the gel were transferred onto polyvinylidene fluoride membrane (Thermo Fischer Scientific, USA). These membranes were blocked with 4% bovine serum albumin for 1 h at 4 ^o^C and then washed, after which they were probed with primary antibodies at 4 ^o^ C overnight. Next, these membranes were incubated with secondary antibodies at 24 ^o^C for 1 h. Finally, protein bands were visualized by Enhanced Chemiluminescence Kits (Millipore, USA).

### 2.9 Transwell invasion assay

HTR-8/SVneo cells (1 x 10^6^ cells/ml) was added onto upper chambers of Transwell inserts plated with Matrigel (Millipore, USA). After these cells were incubated at 37°C for 15 min, 700 μL of DMEM was added to lower chambers, and next cells were incubated for 72 h. After cells which filtered through the upper chambers and Matrigel were removed, Transwell insert was immersed into 70% ethanol for fixation. What followed was that 0.2% crystal violet was added into each well to stain cells in the filter membrane. After crystal violet solution was removed with care, stained cells were visualized and counted under inverted microscope (Olympus, Tokyo, Japan). Cell invasion was calculated based on the protocol reported by Li V. Yang [21].

### 2.10 Tube formation assay

We performed tube formation assay based on the method reported by Rong Shao [22]. After growth factor-reduced Matrigel (BD science, USA) was warmed and distributed in 24-well plates, HTR-8/SVneo cells (2 x 10^4^/ml) were inoculated on the top of Matrigel and then serum-free DMEM was added to each well. Next, they were incubated at 37°C for 24 h. Each well was observed under inverted microscope (Olympus, Tokyo, Japan), and images were taken. We chose 5 random fields in each well and the number of tubules and tubes was counted.

### 2.11 Flow cytometry

To evaluate cell apoptosis and cell cycle, Cell Cylce and Apoptosis Analysis Kit was bought from Beyotime, Shanghai, China. In brief, FITC annexin V and/or propidium were added into each sample to stain cells. What came next was to incubate these cells at about 24 ^o^ C for 10 min and the stained cells would be analyzed by flow cytometry. Data was processed by FlowJo (Three Star, USA).

### 2.12 Statistical methods

Our data was analyzed by operating on GraphPad Prism 8.0 and was displayed in the form of mean ± standard deviation (SD). Two-tailed P < 0.05 was considered as carrying statistical significance. All our experiments were performed five times independently. Student’s t-test and one-way analysis of variance (ANOVA, Bonferroni post hoc test) were adopted in our analysis, depending on the experiment.

## 3. Results

### 3.1 δ-TT induces the apoptosis in trophoblasts and inhibits their proliferation

**Figure 1.**
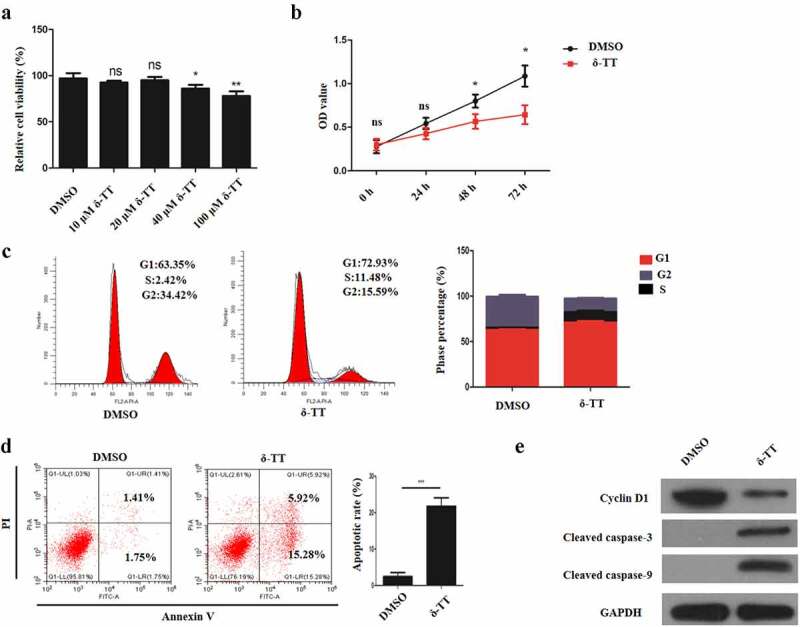
δ-TT induces the apoptosis in trophoblasts and inhibits their proliferation. (a) CCK-8 assay to test cell viability. (b) CCK-8 to examine cell proliferation. (c) Flow cytometry to detect cell cycle. (d) Flow cytometry to detect cell apoptosis. (e) The expression of cyclin D1, cleaved-caspase-3 and cleaved caspase-9 measured by Western blotting. δ-TT, δ-tocotrienol. NS, non-significant, *P < 0.05, **P < 0.01 versus the control group

To assess the effects of δ-TT on trophoblast’s viability and proliferative ability, different concentration of δ-TT was employed to treat trophoblasts, and then cell viability was assessed by CCK-8 assay. As is shown in Figure 1(a), the viability of HTR8/SVneo cells was reduced by δ-TT with its dosage increasing. Then, we chose 100 μM δ-TT for our subsequent experiments. Next, HTR8/SVneo cells were treated with δ-TT for 72 h, and CCK-8 assay and flow cytometry were performed to examine cell proliferation. It was found that δ-TT suppressed their proliferation and shifted more HTR8/SVneo cells to enter G1 phase (Figure 1(b) and 1(c)). Next, the results of flow cytometry and Western blotting showed that δ-tocotrienol triggered trophoblast’s apoptosis and downregulated cyclin D1 activity (Figure 1(d) and 1(e)). Therefore, δ-TT could inhibit the growth of trophoblasts and promote their apoptosis.

### 3.2 δ-TT inhibits the migration, invasion, EMT and angiogenesis in trophoblasts

**Figure 2.**
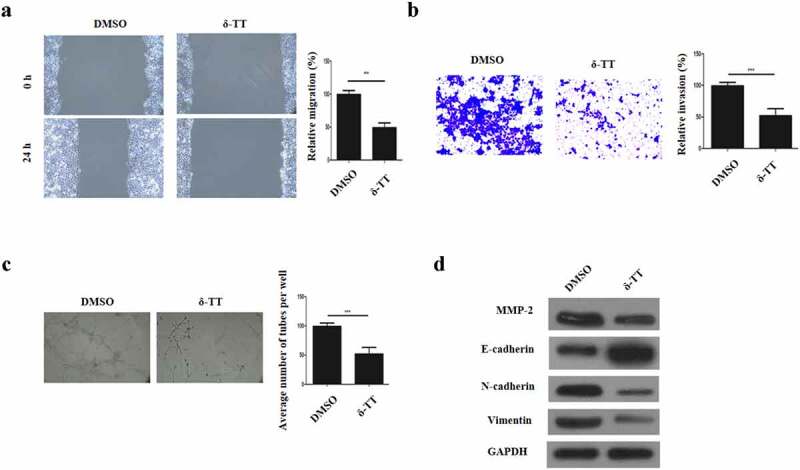
δ-TT inhibits the migration, invasion, EMT and angiogenesis in trophoblasts. (a) Wound healing assay to assess cell migration. (b) Transwell invasion assay to evaluate cell invasion. (c) Tube formation assay to examine angiogenesis. (d) The protein expression of MMP-2 and key epithelial-mesenchymal transition markers. δ-TT, δ-tocotrienol. ***P < 0.001 versus the control group

To evaluate if δ-TT could affect the biological behaviors of trophoblasts, their migration, invasion, vasculogenic activity and EMT was examined. As is displayed in Figure 2(a), trophoblast’s migration was inhibited by δ-TT. Next, through Transwell invasion assay, we found that the invasive ability of trophoblasts was suppressed by δ-TT (Figure 2(b)). To investigate if δ-TT could influence the angiogenesis of trophoblasts, tube formation assay was carried out and the result showed that δ-TT reduced the number of branches and tubes formed by trophoblasts (Figure 2(c)). Next, key EMT markers were evaluated, and we found that δ-TT increased E-cadherin expression and reduced the expression of matrix metalloproteinase-2 (MMP-2), N-cadherin and vimentin (Figure 2(d)). These data suggested that the migration, invasion, EMT and angiogenesis of trophoblasts could be suppressed by δ-TT.

### 3.3 δ-Tocotrienol exerts its toxic effects on trophoblasts via miR-429

**Figure 3.**
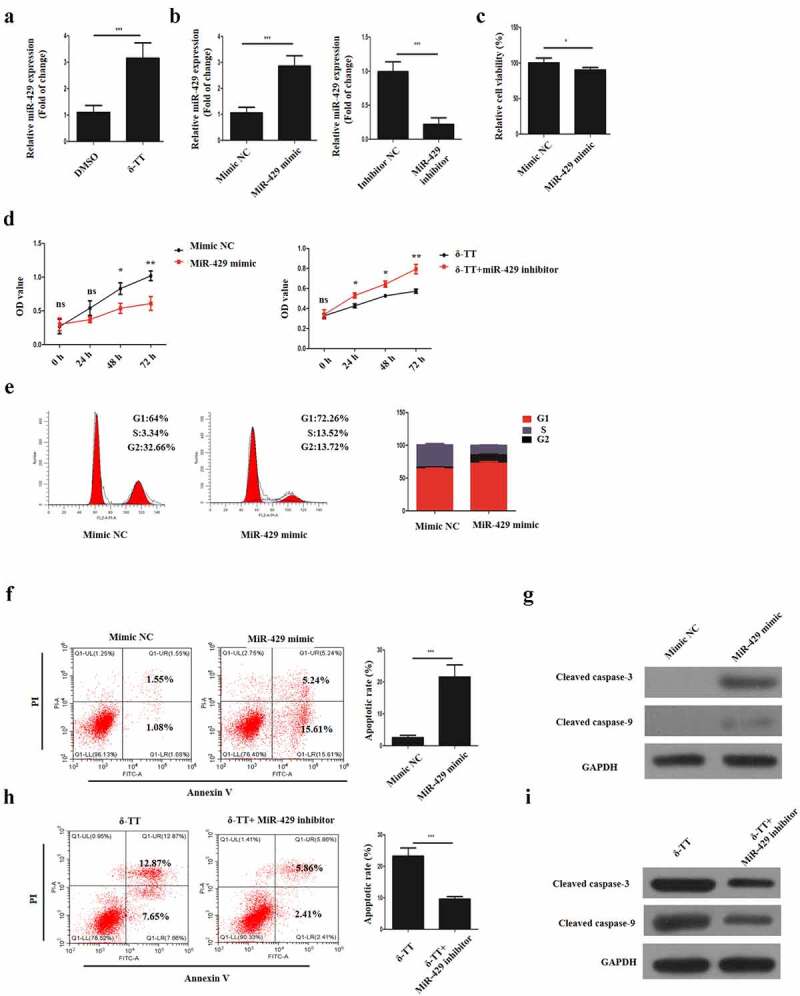
δ-TT exerts its toxic effects on trophoblasts via miR-429. (a) MiR-429 expression following δ-TT treatment. (b) MiR-429 expression after cells were transfected with miR-429 mimic and mimic negative control (NC). (c) Cell viability detected by CCK-8 assay. (d) Cell proliferation measured by CCK-8 assay. (e) Flow cytometry to assess cell cycle. (f and h) Flow cytometry to detect cell apoptosis. (g and i) The expression of cleaved-caspase-3 and cleaved caspase-9 measured by Western blotting. δ-TT, δ-tocotrienol. *P < 0.05, ***P < 0.001 versus the control group

It was found that δ-TT could upregulate miR-429 expression in to promote the apoptosis of breast carcinoma [23], thus we hypothesized that δ-TT might show its toxic impact on HTR-8/SVneo cells with the same mechanism. Then, HTR-8/SVneo cells were subjected to δ-TT and qRT-PCR result showed that δ-TT upregulated miR-429 expression level significantly (Figure 3(a)). To elaborate the role of miR-429 in trophoblasts, HTR-8/SVneo cells were transfected with miR-429 mimic, mimic NC, miR-429 inhibitor and inhibitor NC respectively (Figure 3(b)). Following the transfection, we discovered that upregulating miR-429 expression decreased the viability and growth of HTR-8/SVneo cells (Figure 3(c,e)). Yet, exposing miR-429-downregulated HTR-8/SVneo cells to δ-TT did not impair the viability and proliferative property of HTR-8/SVneo cells (Figure 3(c) and 3(d)). Also, miR-429 led to cell apoptosis (figure 3(f) and 3(g)), whereas treating miR-429-downregulated HTR-8/SVneo cells with δ-TT did not promote the apoptosis of HTR-8/SVneo cells (Figure 3(h) and 3(i)). These data indicate that δ-TT mediates its apoptotic effects on HTR-8/SVneo cells by modulating miR-429.

### 3.4 MiR-429 inhibits the migration, invasion, angiogenesis and EMT in trophoblasts

**Figure 4.**
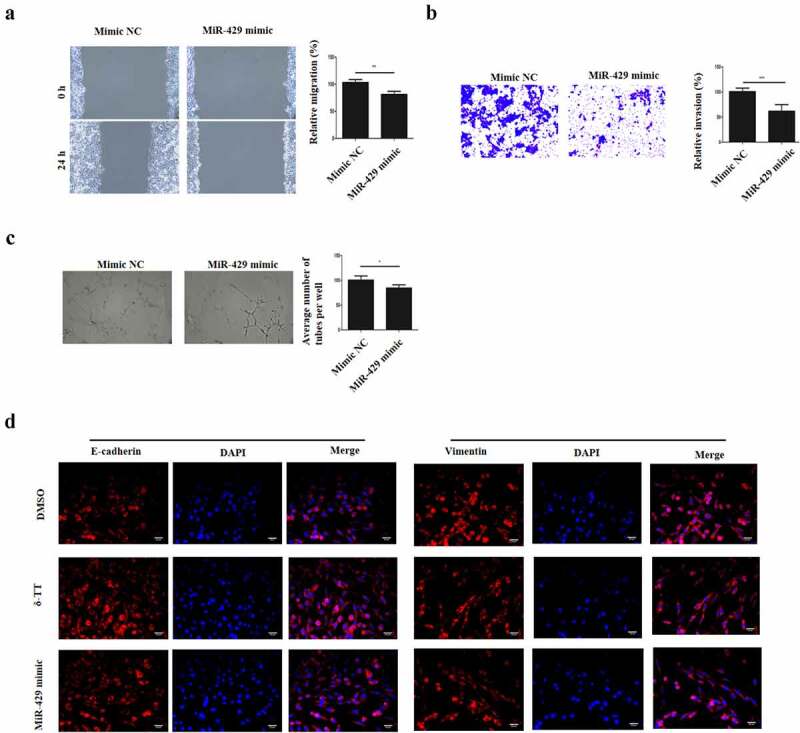
MiR-429 inhibits the migration, invasion, angiogenesis and EMT in trophoblasts. (a) Wound healing assay to assess cell migration. (b) Transwell invasion assay to evaluate cell invasion. (c) Tube formation assay to examine angiogenesis. (d) Immunohistochemistry to assess the expression of E-cadherin and vimentin in trophoblasts. δ-TT, δ-tocotrienol. *P < 0.05, ***P < 0.001 versus the control group

After we found that miR-429 mediated the pro-apoptotic effects on δ-TT, we assumed that miR-429 might negatively regulate the biological behaviors of trophoblasts. Thus, gain-and-loss function experiments were carried out. The migratory property of HTR-8/SVneo cells was reduced by miR-429 (Figure 4(a)). Next, the result of Transwell invasion assay showed that miR-429 reduced the invasion of HTR-8/SVneo cells (Figure 4(b)). Also, miR-429 decreased the vasculogenic activity and EMT in HTR-8/SVneo cells and even EMT (Figure 4(c) and 4(d)). Therefore, the migration, invasion, angiogenesis and EMT of trophoblasts can be inhibited by miR-429.

### 3.5 MiR-429 could bind ZEB1 directly

**Figure 5.**
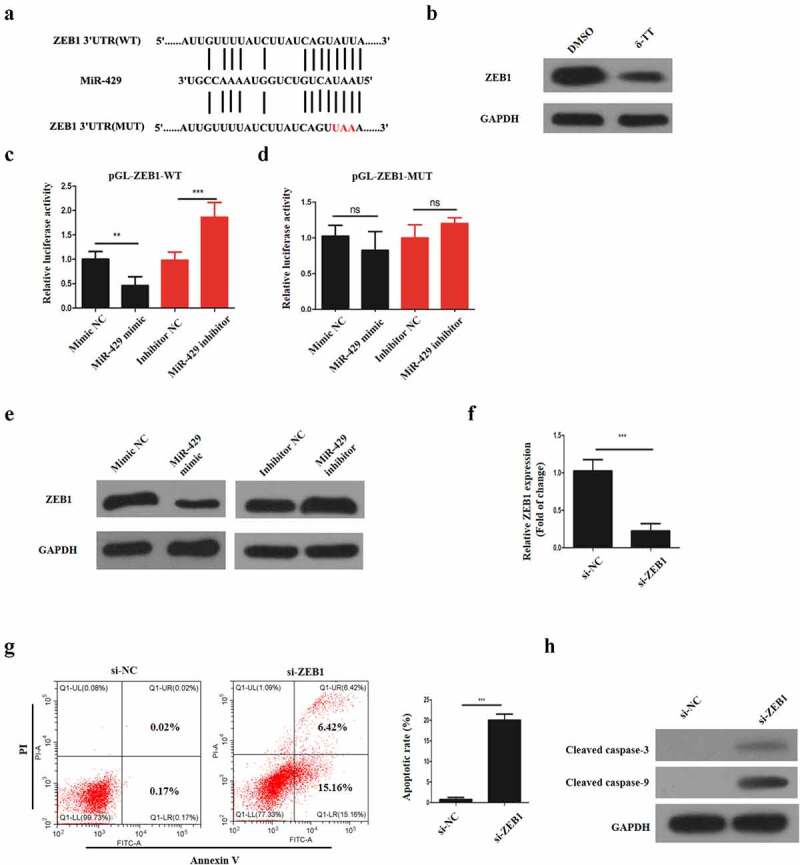
MiR-429 could bind ZEB1 directly. (a) The illustration of the complementary sequence between miR-429 and ZEB1. (b) ZEB1 expression in trophoblasts following δ-TT treatment. (c) The luciferase activity in wild-type luciferase constructs. (d) The luciferase activity in mutant-type luciferase constructs. (e) ZEB1 protein expression. (f) ZEB1 mRNA expression. (g) Flow cytometry to detect cell apoptosis. (h) Western blots for cleaved caspase-3 and cleaved caspase-9. δ-TT, δ-tocotrienol. NS, non-significant, **P < 0.01, ***P < 0.001 versus the control group

**Figure 6.**
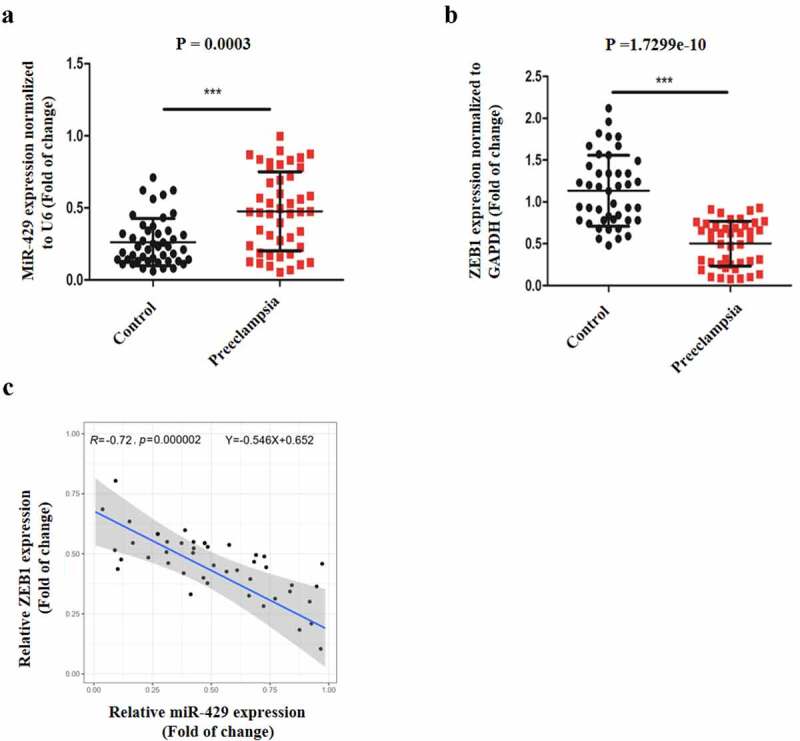
Clinical sample analysis. (a) MiR-429 expression normalized to U6 in normal and preeclamptic placentas. (b) ZEB1 expression normalized to GAPDH in normal and preeclamptic placentas. (c) The relationship between miR-429 and ZEB1 expression in preeclamptic placentas. **P < 0.01, ***P < 0.001 versus the control group

To investigate the underlying mechanism of how miR-329 regulates the biological behaviors of trophoblasts, StarBase was employed to search the potential target of miR-429. It was predicted that ZEB1 might be one of the binding sites of miR-429 (Figure 5(a)). Next, we measured ZEB1 protein expression in trophoblasts, and it was shown that δ-TT downregulated ZEB1 activity (Figure 5(b)). Following that, dual luciferase reporter assay was performed. First, two types of luciferase constructs of ZEB1 were made: pGL3-ZEB1-WT and pGL3-ZEB1-MUT. HTR-8/SVneo cells were co-transfected with pGL3-ZEB1-WT and mimic NC, miR-429 mimic, inhibitor negative control (NC) or miR-429 iinhibitor, respectively In HTR-8/SVneo cells transfected with pGL3-ZEB1-WT, the co-transfection with miR-429 mimic decreased the luciferase activity, whereas the co-transfection with miR-429 inhibitor showed increased luciferase activity (Figure 6(c)). Also, HTR-8/SVneo cells were co-transfected with pGL3-ZEB1α-MUT and mimic negative control (NC), miR-429 mimic, inhibitor negative control (NC) or miR-429 iinhibitor, respectively Yet, in contrast to the control group, transfection with miR-429 mimic did not have any effect on the luciferase activity and the same result was found in the pGL3-IKKα-MUT + miR-429 inhibitor co-transfection group (Figure 6(d)). To confirm the relationship between miR-429 and ZEB1, HTR-8/SVneo cells were transfected with miR-429 mimic and mimic NNC, respectively,and we found that ZEB1 activity was decreased with miR-429 mimic transfection (Figure 6(e)). In addition, HTR-8/SVneo cells were transfected with si-ZEB1 (figure 6(f)), and the knockdown of ZEB1 promoted the apoptosis of these cells (Figure 6(g) and 6(h)). Therefore, we can conclude that ZEB1 is one of the direct binding sites of miR-429.

### 3.6 Clinical sample analysis

Our previous experiments showed that miR-429 upregulation could impair the normal functioning of trophoblasts by binding ZEB1, so we assume that it might regulate PE. Therefore, we performed qRT-PCR to examine miR-429 and ZEB1 expression levels in normal and preeclamptic placentas. As is illustrated in Figure 6(a), miR-429 expression in preeclamptic placentas was upregulated in comparison with that in normal placentas. Also, ZEB1 expression levels in preeclamptic placentas were much lower, in contrast to normal placentas (Figure 6(b)). Additionally, in preeclamptic placentas, there was a negative correlation between miR-429 and ZEB1 expression (Figure 6(c)). Thus, miR-429 might play a role in the pathogenesis of PE.

## 4. Discussion

Many studies have demonstrated that high-dose vitamin E intake may pose a risk to people’s health. For example, high-dose vitamin E intake could raise all-cause mortality in patients with chronic diseases and even have a negative impact on conceptus [24]. There are controversies as to the beneficial effects of vitamin E on pregnancy and PE [25], so this research focused on exploring the influence of δ-TT (an isomer of vitamin E) on trophoblasts. Our initial experiments showed that δ-TT could decrease the viability of trophoblasts and result in the apoptosis of trophoblasts. It is noteworthy that δ-TT has been proven to promote the apoptosis in many types of cancers [19,26,27], which are consistent with our discovery in trophoblasts. Therefore, δ-TT might carry teratogenic risk in pregnancy.

Furthermore, our research has shown that δ-TT can suppress the migration, invasion, EMT and vasculogenic activity in trophoblasts. The migration, invasion, EMT and angiogenesis are important functions of trophoblasts during pregnancy, as they need to invade decidua to remodel spiral arteries and establish the circulatory network between mother and fetus [28–30]. Also, trophoblasts differentiate from an epithelial phenotype into a mesenchymal phenotype when they invade at the maternal-fetal interface and EMT is a vital process of trophoblast invasion [31]. Trophoblastic dysfunction can lead to placental malfunction, spontaneous abortion and even PE [32]. Considering important biological functions of trophoblasts, our findings support the potential detrimental impact of δ-TT on pregnancy and PE.

Mechanistically, we discovered that miR-429 expression was upregulated by δ-TT to mediate the pro-apoptotic, anti-migratory, anti-invasive and anti-angiogenic impact on trophoblasts, as exposing miR-429-downregulated trophoblasts to δ-TT has been demonstrated to abrogate the pro-apoptotic effects of δ-TT on trophoblasts. In addition, upregulating miR-429 expression can promote the apoptosis of trophoblasts and impede their migration, invasion and angiogenesis. These data have revealed that it is miR-429 that mediates the toxic influence of δ-TT on trophoblasts. The biological role of miR-429 has been well elucidated in multiple cancers and pregnancy. For example, miR-429 overexpression can inhibit migration and invasion in breast cancer and colorectal carcinoma [33,34]. Interestingly, miR-429 participates in the regulation of angiogenesis in glioma cells [35]. During the third trimester of pregnancy, miR-429 expression has been found to be downregulated significantly [36]. Therefore, miR-429 acts as a central role to mediate the toxic influence of δ-TT and increasing miR-429 expression can be detrimental to pregnancy and even deteriorate PE.

To explore the deeper mechanism of how miR-429 influences the biological behaviors of trophoblasts, StarBase was used to screen the potential genes, and we found that miR-429 could bind ZEB1 directly. It is of note that this interaction was reported in renal cell carcinoma and epithelial ovarian cancer [37,38]. Our experiments confirmed this interaction and have revealed that δ-TT can suppress the activity of ZEB1 in trophoblasts. It has been demonstrated that ZEB1 is key to the proliferation, migration, invasion and survival of trophoblasts, as the knockdown of ZEB1 in trophoblasts can reduce the growth, migration, invasion and EMT in trophoblasts via Akt signaling pathway [39,40]. Also, inhibiting ZEB1 activity can promote cell apoptosis [41,42], and it serves to promote vasculogenic activity by upregulating the expression of vascular endothelial growth factor A (VEGFA) [43,44]. In addition, our clinical specimen analysis shows that miR-429 has been discovered to be upregulated in preeclamptic placentas and that its expression positively correlated with ZEB1 expression. Therefore, we thought that inhibiting ZEB1 activity could exacerbate preeclampsia, as it might cause the dysfunction of trophoblasts.

However, there are limitations in this research. First, we should establish a rat PE model to study if δ-TT could increase the severity of PE. Second, we should obtain patients’ blood samples and analyze their plasma δ-TT concentration to further validate our research findings.

## Conclusion

In summary, we have found that δ-TT decreases the viability, growth, migration, invasion, EMT and angiogenesis in trophoblasts through miR-429/ZEB1 axis, and miR-429 expression is upregulated in preeclamptic placentas. These results indicate that δ-TT might not be suitable to be used in pregnancy and can worsen PE. Also, targeting miR-429 expression might provide a new approach to the treatment of PE.

## Data Availability

The datasets used and/or analyzed during the present study are available from the corresponding author on reasonable request.

## Highlights

1. δ-Tocotrienol can promote the apoptosis of trophoblasts.
2. The viability, migration, and EMT of trophoblasts can be inhibited by δ-tocotrienol.
3. δ-Tocotrienol modulates trophoblast’s biological behavior via miR-429/ZEB1 axis.
4. MiR-429 expression is upregulated in preeclamptic placenta.
